# Recipients of COVID-19 vaccines face challenges of SARS-CoV-2 variants

**DOI:** 10.7150/ijbs.72424

**Published:** 2022-07-11

**Authors:** Tianhong Li, Kathy Qian Luo

**Affiliations:** 1 Faculty of Health Sciences, University of Macau, Macao SAR, China; 2 Ministry of Education-Frontiers Science Center for Precision Oncology, University of Macau, Taipa, Macao SAR, China

**Keywords:** COVID-19, SARS-CoV-2 virus, Vaccines, Viral variants, booster vaccination

## Abstract

The coronavirus disease 19 (COVID-19) has been rampant since 2019, severely affecting global public health, and causing 5.75 million deaths worldwide. So far, many vaccines have been developed to prevent the infection of SARS-CoV-2 virus. However, the emergence of new variants may threat vaccine recipients as they might evade immunological surveillance that depends on the using of anti-SARS-CoV-2 antibody to neutralize the viral particles. Recent studies have found that recipients who received two doses of vaccination plus an additional booster shoot were able to quickly elevate neutralization response and immune response against wild-type SARS-CoV-2 virus and some initially appeared viral variants. In this review, we assessed the real-world effectiveness of different COVID-19 vaccines by population studies and neutralization assays and compared neutralization responses of booster vaccines *in vitro*. Finally, as the efficacy of COVID-19 vaccine is expected to decline over time, continued vaccination should be considered to achieve a long-term immune protection against coronavirus.

## Introduction

COVID-19, caused by severe acute respiratory syndrome coronavirus 2 (SARS-CoV-2), has ramparted the whole world since 2019, severely affecting global public health, causing social disruption and significant economic losses. As of February 2022, there were more than 396 million confirmed cases and 5.75 million deaths worldwide 1. With global spread of SARS-CoV-2, mutations are accumulating during replication, leading to viral variants that increase transmissibility, virulence or reduce effectiveness of vaccines and treatments 2.

SARS-CoV-2 contains four major structural proteins (spike protein, membrane protein, envelope protein, and nucleocapsid protein), which are encoded by individual open reading frames (ORFs) 3, 4. Spike protein is a glycoprotein located on the surface of SARS-CoV-2 viral particle. This protein can be cleaved into two domains: S1 and S2. S1 domain includes the N-terminal domain (NTD) and receptor-binding domain (RBD), and RBD can help coronavirus bind angiotensin-converting enzyme 2 (ACE2) located at the surface of the host cells such as endothelial cells 5, 6. Studies showed that the binding free energy (BFE) between RBD and ACE2 was positively correlated with viral infectivity 7, 8. The function of S2 domain can help the viral particle to enter the host cells via membrane fusion 9. Among the three major structure proteins of SARS-CoV-2: spike, membrane and nucleocapsid, the gene encodes spike protein has the highest frequency of mutation especially in the RBD region that is used to design the currently used anti-COVID-19 vaccine 10. Many antibody therapeutics and vaccines have been designed to target the spike protein based on the initially discovered viral strain that caused COVID-19 in early 2020. The accumulation of these spike gene mutations in the new coronavirus is more likely to affect the effectiveness of developed vaccines against these new viral variants 11.

However, during the global spread of SARS-CoV-2, many new variant strains of this coronavirus have been isolated which may be formed due to the accumulation of numerous mutations in the past two years 12. Many mutations in spike protein, which strengthen the binding between the spike protein and ACE2, have been shown to affect the effectiveness of the existing anti-COVID-19 vaccines and monoclonal antibodies 13. The mutagenesis has three mechanisms: molecular scale, organism scale, and population level. The first two mechanisms provide numerous candidate mutations in the SARS-CoV-2 genome. The population-level mechanism determines what mutations are predominant by natural selection that have two pathways of infectivity and vaccine resistance 8. In the summer of 2020, Michigan State University established a model of infection based on natural selection and predicted that SARS-CoV-2 was more infectious on the mutations of residues 452, 489, 500, 501, and 505 in the RBD region, after computing the BFE changes of possible mutations by integrating genotyping, deep learning, biophysics, and mathematics 14. The result was confirmed by pandemic SARS-CoV-2, in the last two years. Recently, vaccine-resistant mutations that are emerged after vaccinations, have been confirmed. Vaccine-resistant mutations with negative BFE changes, that disrupted the binding between the spike protein and antibodies, have been observed frequently, such as Y449S and Y449H, after many people in many countries were vaccinated in high rates 8. The trend of increasing frequency of vaccine-resistant mutations correlated strongly with the proportion of fully vaccinated in Europe and America. Mutations in vaccine-resistant pathways also reduce the effectiveness of vaccines and antibody therapies, suggesting that COVID-19 will be a prolonged pandemic.

As of February 2022, World Health Organization (WHO) listed five designated variants of concern (VOCs) for coronavirus: Alpha (B.1.1.7, earliest documented in the UK in September 2020), Beta (B.1.351, earliest documented in South Africa in May 2020), Gamma (P.1, earliest documented in Brazil in November 2020), Delta (B.1.617.2, earliest documented in India in October 2020), and Omicron (B.1.1.529, earliest documented in multiple countries in November 2021) (**Table 1**). WHO has also listed two designated variants of interest (VOIs): Lambda (C.37, earliest documented in Peru in December 2020) and Mu (B.1.621, earliest documented in Colombia in January 2021) 15.

Vaccines are constantly been developed to achieve immune responses to SARS-CoV-2 virus 16. However, vaccine development is a long-lasting and challenging process, taking years to complete. Currently, there are 195 vaccines in preclinical development and 142 vaccines in clinical trials 17. As of 12 January 2022, 10 vaccines are listed under WHO Emergency Use Listing Procedure (EUL), which include four types: mRNA vaccines, viral vector vaccines, recombinant spike vaccines and inactivated vaccines. These vaccines are being widely used around the world to achieve immune protection against SARS-CoV-2. By February 2022, more than 3.7 billion people were vaccinated with at least 1 dose worldwide, about 1/4 of the total population. There are 2.2 billion people that were fully vaccinated worldwide 18. Many countries are working together to roll out a COVID-19 vaccine globally.

But the emergence of these new, potentially more infectious SARS-CoV-2 variants challenges vaccine protection 19. Based on the changes of BFE, it was found that some mutations made SRAS-COV-2 more infectious. Three key RBD mutations sites of N501, L452 and Y505H were proved in VOCs that promote transmission and break through the protection of vaccines (**Table 1**). The Delta and Alpha variants documented in 2020 were found to be more infectious, leading to a global pandemic. The Omicron variant is currently the most dominant pandemic strain, which accounted for 85% of new cases in January of 2022 20. The Omicron variant, designated by WHO as VOC within two days, has three lineages, BA.1, BA.2, and BA.3 (**Table 1**) 21. BA.1 contains up to 32 mutations in the spike gene and 15 of these mutations clustered in the region encoding RBD strengthen infectivity and disrupt monoclonal antibodies 22-27. Spike proteins are critical for viral entry and the main target of neutralizing antibodies. The Omicron variant has thus raised concerns that this viral stain may escape from protection conferred by vaccines. Recently, Omicron BA.2, which was more infectious than BA.1 based on changes of BFE and could lead to breakthroughs in vaccines, is replacing the BA.1 in China 28. The lack of clinical data on the effectiveness of vaccines against new variants creates an uncertainty that could prolong the duration of the pandemic. We need to understand the effectiveness of COVID-19 vaccines, especially against VOCs.

Therefore, the purpose of this article is to review the recent studies on the effectiveness of vaccines against different SARS-CoV-2 variants. We hope this review will provide an overview on the effectiveness of various types of vaccines, which will contribute to the development of new treatments and new vaccines.

## Effectiveness of clinical vaccines against VOCs

VOCs were able to break through the protection of vaccines, evade immunological surveillance and escape neutralizing antibodies after vaccination 23, 29-33. So far, the Alpha variant has been found to have limited effect on the efficacy of the vaccine. Numerous data and research have demonstrated that neutralization response and vaccine-induced immune protection declined against the Omicron variant compared to wild-type virus and other VOCs (**Table 2**). The emerging evidence supports that the neutralizing antibodies against SARS-CoV-2, which can block the interaction between the spike protein with ACE2, play an important role in protective immunity after vaccination or infection. B and T lymphocytes also contribute to the protective effect against symptomatic infection. Several monoclonal antibody-based therapeutics have been approved to treat patients with SARS-CoV-2 34. However, the anti-Omicron activity of many monoclonal antibodies was diminished 23, 25, 35, 36. Next, this review will discuss the efficacy of several commonly used vaccines against the coronavirus variants from published preclinical and clinical data in population studies and laboratory tests.

### mRNA vaccine

The mRNA-1273 (Moderna) vaccine was co-developed by researchers at the National Institute of Allergy and Infectious Disease in USA. In population-based studies, effectiveness of mRNA-1273 vaccine after full doses was approximately 73% against infection of the Alpha variant, but 61% against infection of the Beta variant 37, 38 (**Table 2**). Only 55% of serum samples, randomly selected from participants in the Coronavirus Efficacy (COVE) phase 2 and phase 3 trials, were detected to have neutralization response against the Omicron variant by geometric mean neutralizing titers (GMTs). Together, neutralization titers were 34.8-fold lower against the Omicron variant than the wild-type virus 33. The effectiveness against omicron infection was approximately 44% at two weeks after vaccination, but 5.9% after 9 months 39.

In population-based studies, researchers in the UK found that vaccination with BNT162b2 (Pfizer-BioNTech) vaccine or AZD1222 (AstraZeneca) vaccine decreased the risk of infection with the Alpha variant, but the risk of infection with the Delta variant increased relative to the Alpha variant 40. The effectiveness of BNT162b2 vaccine against infection was approximately 94% against the Alpha variant, 88% against the Delta variant 41, 72% against the Beta variant 42 and 70% against the Omicron variant 43 (**Table 2**). The neutralization titers of serum samples also verified these results against various viral strains (wild-type virus ˃ Delta variant ˃ Beta variant ˃ Omicron variant) and the neutralization efficiency with two doses of BNT162b2 vaccine against Omicron variant was lower (by a factor of about 20) than wild-type virus 43, 44.

In summary, these reports showed that after administration of aforementioned vaccines, the antibody titers against Omicron variant were lower than those against the wild-type virus and some stains of VOCs, especially the Omicron variant, may escape mRNA vaccines-induced immune protection.

### Viral vector vaccines

AZD1222 (also known as ChAdOx1 nCoV-19) consists of a replication-deficient chimpanzee adenoviral vector containing the sequence of SARS-CoV-2 spike gene. The efficacy after two-doses of AZD1222 vaccination was approximately 70% against symptomatic infection of the Alpha variant 45 (**Table 2**). Likewise, a study by University College London Hospitals and the Francis Crick Institute in London showed that 37% serum samples with two-doses of AZD1222 vaccination were detected with quantifiable neutralizing antibody titer against the Omicron variant, which was lower than the detected rate against the Alpha and the Delta variants 46. The neutralization titers of serum samples against different viral strains ranged in the following order: Alpha ˃ Delta ˃ Omicron. The research by College of Life Science and Technology, Beijing University of Chemical Technology also found that serum samples from some recipients of two-dose AZD1222 did not exhibit any detectable titer of neutralizing antibody against the Omicron variant and those data suggested that the Omicron variant is antigenically more different from the wild-type virus than the Beta and Delta variants 47. So, those results showed that recipients who received viral vector vaccines (i.e. AZD1222) displayed a decreased neutralization response and immune response relative to wild-type virus than to some of VOCs, especially the Omicron variant.

### Recombinant spike vaccines

ZF2001, a recombinant dimeric RBD protein vaccine, induces RBD-directed neutralizing response, and is undergoing phase 3 clinical trials 48-50. All the serum samples of ZF2001 recipients displayed positive neutralization response against wild-type virus and ZF2001 also preserved the neutralizing activity against the Delta variant 51. However, it was almost impossible for ZF2001 to neutralize the Omicron variant 52.

### Inactivated vaccines

SARS-CoV-2 inactivated vaccines elicit immune response directed against the entire viral particle instead of only targeting the spike protein or RBD of the virus 53. After 4 months, neutralizing antibodies against Gamma and Delta variants were limitedly detected with two-dose CoronaVac (Sinovac) 54. Among the vaccine recipients, all serum samples were negative for both Omicron and Delta variants 55. The third dose of CoronaVac was found to stimulate cross-protective B and T cell responses against variants 30, 56.

BBIBP-CorV (Sinopharm, Beijing CNBG), authorized by the Hungarian National Drug and Food Evaluation Authority, contains SARS-CoV-2 inactivated in Vero cells. Ninety percent of participants were below the age of 50 and they were detected to have RBD-binding antibody after two doses of BBIBP-CorV vaccination 57. Neutralization titer was decreased against the Beta variant, a greater degree was found with the Delta variant. RBD binding antibodies of the serum samples were also decreased against the VOCs 58. It was also reported that the neutralizing activity against wild-type virus was 80% after two doses of inactivated BBIBP-CorV and only 10% serum samples showed successful neutralization against the Omicron variant. Neutralization titer against Omicron variant was also significantly reduced relative to wild-type virus in serum samples recovered from Delta variant infection 35.

However, the BBIBP-CorV vaccine was effective in generating cross-protective B and T cell responses to prevent infection of other variants, which were mutated in spike protein 59, 60. Those cross-protective responses have also been found after receiving vaccination of mRNA-1273 and BNT162b2 mRNA 61, 62. It thus suggests that the inactivated vaccine can generate cell-mediated immune responses against other variants.

## Effectiveness of vaccine booster against VOCs

Although the efficiency of vaccination could be decreased over time, some studies have shown that both inactivated vaccines and mRNA vaccine booster significantly increased the neutralization titers to wild-type virus and other variants, restored efficiency of vaccine and reduced virus transmission 30, 36, 63-65.

It has been observed that the neutralization efficiency against the wild-type virus was decreased by 7.6 times after 6 months of vaccination with a second dose of the mRNA-1273 vaccine 33. The results obtained from 239 COVID-19 vaccinees showed that booster mRNA vaccines (mRNA-1273 and BNT162b) increased neutralizing antibody response compared to two-dose mRNA vaccines, especially against the Omicron variant. After the third dose of mRNA-1273 vaccine, neutralization against the Omicron variant was detected in all serum samples, but not in some serum samples after two-dose mRNA-1273 vaccine 30, 33. Data from 49 states of U.S. in December 2021 showed that receipts of BNT162b2 vaccine booster was protective against the Omicron and Delta variants, while there was less protection for the Omicron variant than for the Delta variant 63. It was also reported that the third dose of BNT162b2 vaccine was 100 times more effective in neutralizing the Omicron variant than the two-dose vaccination 44.

The neutralization titers against the Omicron variant and wild-type virus were elevated after the third inactivated vaccine 35. The neutralization titer of BBIBP-CorV/ZF2001 heterologous booster group was higher relative to homologous booster group 66. Both homologous and heterologous inactivated vaccine booster increased neutralizing antibodies and improved the protection against the Omicron variant 35. Results demonstrated that the ZF2001 booster was more efficient against SARS-CoV-2 variants that have mutations in NTD of the spike, such as the Delta variant 54. The study from Fudan University found that after BBIBP-CorV booster, the levels of antibody in the blood samples were increased, and humoral immune responses of spike-specific memory B and T cells were quickly elevated 67. Therefore, it is very necessary to carry out COVID-19 vaccine booster to restore the efficiency of vaccine and limit virus transmission.

## Conclusion

During the current pandemic, SARS-CoV-2 variants are continuously selected, which can evade immunological surveillance and promote transmission. Vaccines can loss immunity to SARS-CoV-2 variants due to the mutations in the spike proteins. The emergence of new variants threat vaccine recipients and researchers are finding new solutions to attenuate immune evasion.

Interestingly, recipients who received an additional booster vaccine after two doses quickly elevated neutralization response and immune responses of spike-specific memory B and T cells and similar results were also observed in rhesus macaques 68. However, the efficiency of vaccination decreased over time. For example, even after the second dose of the mRNA-1273 vaccination, the neutralization efficiency against the wild-type virus was decreased by 7.6 times after 6 months 33. Similarly, after a third booster dose of mRNA-1273 vaccine, the neutralization efficacy against the Omicron variant was decreased by 6.3 times after five months. Therefore, continued vaccination even after the vaccine booster should be considered to achieve long-term immune protection against new emerging variants of SARS-CoV-2.

## Figures and Tables

**Table 1:** Variants of SARS-CoV-2 and mutations in RBD.

Variants		Mutations
Alpha (B.1.1.7)		E484K; S494P; N501Y
Beta (B.1.351)		K417F; E484K; N501Y
Gamma (P.1)		K417F; E484K; N501Y
Delta (B.1.617.2)		K417T; L452R; T478K
Omicron (B.1.1.529)	BA.1	G339D; S371L; S373P; S375F; K417N; N440K; G446S; S477N; T478K; E484A; Q493R; G496S; Q498R; N501Y; Y505H
	BA.2	G339D; S371L; S373P; S375F; T376A; D405N; R408S; K417N; N440K; S477N; T478K; E484A; Q493R; Q498R; N501Y; Y505H
	BA.3	G339D; S371L; S373P; S375F; D405N; K417N; N440K; G446S; S477N; T478K; E484A; Q493R; Q498R; N501Y; Y505H

**Table 2:** Efficacy and neutralization efficiency of different vaccines

Outcome	Wild type	Alpha	Delta	Beta/Gamma	Omicron
**Efficacy against infection %**					
mRNA-1273 (Moderna)	94	73	70	61	44
BNT162b2 (Pfizer-BioNTech)	95	94	88	72	70
AZD1222 (AstraZeneca)	74	70	60	64	N/A
